# Heteromerization Modulates mu Opioid Receptor Functional Properties *in vivo*

**DOI:** 10.3389/fphar.2018.01240

**Published:** 2018-11-13

**Authors:** Muzeyyen Ugur, Lyes Derouiche, Dominique Massotte

**Affiliations:** Centre de la Recherche Nationale Scientifique, Institut des Neurosciences Cellulaires et Intégratives, Université de Strasbourg, Strasbourg, France

**Keywords:** mu opioid receptor, heteromer, GPCR, delta opioid receptor, morphine, hypertension, addiction, pain

## Abstract

Mu opioid receptors modulate a large number of physiological functions. They are in particular involved in the control of pain perception and reward properties. They are also the primary molecular target of opioid drugs and mediate their beneficial analgesic effects, euphoric properties as well as negative side effects such as tolerance and physical dependence. Importantly, mu opioid receptors can physically associate with another receptor to form a novel entity called heteromer that exhibits specific ligand binding, signaling, and trafficking properties. As reviewed here, *in vivo* physical proximity has now been evidenced for several receptor pairs, subsequent impact of heteromerization on native mu opioid receptor signaling and trafficking identified and a link to behavioral changes established. Selective targeting of heteromers as a tool to modulate mu opioid receptor activity is therefore attracting growing interest and raises hopes for innovative therapeutic strategies.

## Introduction

The mu opioid (mu) receptor is a G protein-coupled receptor (GPCR) that neuromodulates several physiological functions, in particular nociception (Kieffer and Evans, 2009). This receptor also mediates the reinforcing properties of natural stimuli. In addition, mu receptors are the primary molecular target of opioid drugs used in the clinic (e.g., morphine, codeine, oxycodone, fentanyl, tramadol), and are responsible for their analgesic properties but also for the side effects associated with their acute (e.g., respiratory depression, nausea, dizziness, sedation, constipation) (Kieffer, 1999) and chronic use (tolerance, hyperalgesia, and physical dependence) (Matthes et al., 1996; Williams et al., 2013). Moreover, mu receptors mediate opioid rewarding and euphoric properties that underlie their abuse potential (Matthes et al., 1996). The latter is at the root of the epidemic that has developed in North America upon misuse and/or abuse of prescription opioid drugs after an initial therapeutic use or in patients that self-medicate (Vowles et al., 2015). It underscores the need for designing effective opioid analgesics devoid of side effects and has prompted considerable efforts to better understand the molecular and cellular mechanisms underlying mu receptor activity. In this context, functional consequences elicited by physical association of the mu receptor with another GPCR attracted attention. Here, we review evidence of molecular, cellular, and behavioral modulation induced by mu receptor heteromerization in native tissue.

## Mu receptor heteromers in native tissue

Heteromers involving the mu receptor have been extensively studied in heterologous systems (Fujita et al., 2014a). Receptor physical proximity has now been established in native tissue for several receptor pairs using receptor co-immunoprecipitation, co-localization by electron microscopy or *in situ* proximity ligation assay (PLA), and/or disruption of physical contact by an interfering peptide. The use of interfering peptide and/or mice deficient for one receptor also significantly contributed to demonstrate the specificity of the functional changes associated with heteromer formation and to establish a causal link with behavioral outputs. In native tissue, the mu receptor heteromerizes with the delta opioid (delta) or kappa opioid (kappa) receptors or with the non-opioid receptors ORL1, cannabinoid CB_1_, galanin Gal1, adrenergic α_2a_, somatostatin sst_2_, dopamine D_1_, chemokine CCR5, and vasopressin V_1b_. Association between the mu receptor splice variant MOR_1D_ and the gastrin-releasing peptide receptor (GRPR) has also been reported as well as mu physical proximity with the ion channel NMDA (Table 1). Heteromerization with a Gα_i/o_ coupled GPCR is thus the most frequently reported to date but association with the Gα_q_ coupled GRPR and vasopressin V_1B_ receptors or the Gα_s_ coupled dopamine D_1_ receptor indicates no specific requirement. Additional heteromers likely exist *in vivo* since mu receptor heteromerizes with serotonin 5HT_1a_ (Cussac et al., 2012), neuropeptide FF NPFF_2_ (Roumy et al., 2007), melanocortin MC3 (Rediger et al., 2009), neurokinin NK1 (Pfeiffer et al., 2003), and possibly, dopamine D4 (Qian et al., 2018) receptors in co-transfected cells, and neuronal co-localization with chemokine CXCR4 (Patel et al., 2006; Heinisch et al., 2011), metabotropic glutamate mGluR5 (Schröder et al., 2009) and serotonin 5HT_2a_ (Lopez-Gimenez et al., 2008), and dopamine D_4_ (Rivera et al., 2017) receptors has been reported (see also http://www.gpcr-hetnet.com for further information on the GPCR interaction network, and interacting or non-interacting receptor pairs; Borroto-Escuela et al., 2014).

**Table 1:** Identification, properties, and functional outcome of native heteromers involving the mu opioid receptor.

**Receptor pair**	***In vivo***** physical proximity**			**Specific properties of native heteromers**			
	**Tissue**	**Technique**	**References**	**Ligand binding, receptor signaling and trafficking**	**Tissue**	**Functional outcome**	**References**
**MU HETEROMERS INVOLVING ASSOCIATION WITH A GI/O COUPLED RECEPTOR**							
Mu-delta	Mouse brain, SC, DRG	Co-IP Disruptive peptide	Xie et al., 2009; Kabli et al., 2013; Erbs et al., 2015	Reciprocal positive crosstalk upon co-activation with an agonist, inverse agonist or antagonist for the other receptor (positive binding cooperativity, increased Gα signaling)	SKNSH, SC VTA		Gomes et al., 2000, 2004, 2011; Margolis et al., 2017
	Increased by morphine in Selected brain areas	Specific mu-delta antibody	Gupta et al., 2010			
				Synergy upon co-activation in chronic morphine treated rats	RVM	Increased analgesia	Zhang and Pan, 2010
				Synergy upon co-activation in chronic inflammatory condition	RVM	Increased analgesia	Sykes et al., 2007
				Mu-delta surface expression	DRG, SC	Increased analgesia	Walwyn et al., 2009; Xie et al., 2009
				Disruption mu-delta heteromer	SC, DRG	Increased Morphine tolerance	Xie et al., 2009; He et al., 2011
				Mu-delta co-internalization (UFP-512, CYM51010)	Striatum, hippocampus	Anxiolytic, anti- depressive, analgesic, decreased morphine tolerance and dependence	Gomes et al., 2013; Kabli et al., 2013; Derouiche et al., 2018
				Increased β-arrestin signaling	SKNSH cells		Rozenfeld and Devi, 2007
				No uncoupling from Gz after chronic morphine	Striatum, hippocampus		Kabli et al., 2014
				DAMGO induced delta recycling to plasma membrane after chronic morphine	DRG		Ong et al., 2015
Mu-kappa	Rat SC proestrous females	Co-IP	Chakrabarti et al., 2010	Co-activation morphine/dyn1-17 induced synergy		Increases morphine analgesia females	Chakrabarti et al., 2010; Liu N. J. et al., 2011
Mu-ORL1	DRG	Co-IP	Evans et al., 2010	Co-activation induced negative crosstalk on ORL1 signaling	Neuroblastoma	Nociception	Mandyam et al., 2002
Mu-CB_1_	Rat striatum	Electron microscopy	Rodriguez et al., 2001	Co-activation induced bidirectional negative crosstalk, decreased mu agonist binding Bidirectional cross antagonism (Nacc)	SKNSH, striatum Mu KO mice CB_1_ KO mice CB_1_ antagonist	Neuritogenesis Social play	Vaysse et al., 1987; Rios et al., 2006 Manduca et al., 2016
Mu-Gal1	Mouse VTA	Disruptive peptide	Moreno et al., 2017	Co-activation induced negative crosstalk Cross-antagonism on Gal1 signaling	VTA	Opioid drug reward	Moreno et al., 2017
Mu-α_2a_ adrenergic	Rat NTS Increased expression in hypertensive rats	Co-IP PLA	Sun et al., 2015	Opiate induced increased co-expression Co-activation induced negative crosstalk receptor co-internalization	RVM Primary SC neurons DRG	Hypertension	Sun et al., 2015 Jordan et al., 2003 Tan et al., 2009
Mu-sst2	Human pancreatic cancer cells	Co-IP FCS	Jorand et al., 2016	Co-activation increased β-arrestin signaling, decreased EMT	Pancreatic cancer cell line	Increased cancer metastatis	Jorand et al., 2016
Mu-CCR5	Human and monkey PBMC	Co-IP	Suzuki et al., 2002	Negative crosstalk Cross-antagonism	CCR5 KO mice CCR5 antagonist	Decreased nociception HIV infection	Lee et al., 2013 Szabo et al., 2002
**MU HETEROMERS INVOLVING ASSOCIATION WITH A Gs COUPLED RECEPTOR**							
Mu-D_1_	Mouse striatum mPFC	Co-IP Co-localization	Tao et al., 2017	Cross-antagonism	D_1_ KO mice D_1_ antagonist	Opiate locomotor sensitization	Tao et al., 2017
**MU HETEROMERS INVOLVING ASSOCIATION WITH A Gq COUPLED RECEPTOR**							
Mu-V_1b_	Mouse RVM	ISH Truncated V_1b_ receptor	Koshimizu et al., 2018	Increased β-arrestin signaling	RVM	Enhanced morphine tolerance	Koshimizu et al., 2018
MOR1D-GRPR	Mouse SC	Co-IP Disruptive peptide	Liu X. Y. et al., 2011	Positive crosstalk on GRPR signaling	SC	Morphine induced itch	Liu X. Y. et al., 2011
**MU HETEROMERS INVOLVING ASSOCIATION WITH AN ION CHANNEL**							
Mu-NMDA	Mouse PAG	Co-IP	Rodríguez-Muñoz et al., 2012	Positive crosstalk on mu receptor and negative crosstalk on NMDA CAMKII pathway	PAG	Decreased morphine analgesia and increase morphine tolerance	Rodríguez-Muñoz et al., 2012

*Co-IP, Co-immunoprecipitation; DRG, Dorsal Root Ganglia; PAG, Periaqueductal Gray; PBMC, peripheral blood mononuclear cells; PLA, Proximity Ligation Assay; RVM, Rostral Ventral Medulla; SC, Spinal Cord; VTA, Ventral Tegmental Area*.

Expression of native heteromers is dynamic. Chronic morphine treatment enhances mu-delta heteromer density in brain regions associated with the reward pathway (Gupta et al., 2010). Concomitant increase in delta receptor localization at the cell surface is observed and is mu receptor dependent (Gendron et al., 2015; Ong et al., 2015; Erbs et al., 2016). Heteromers form intracellularly in native tissue. In the mouse dorsal root ganglia (DRG), mu and delta opioid receptors associate in the endoplasmic reticulum (ER), which requires phosphorylation of the delta receptor at threonine 161 by the cdk5 kinase (Walwyn et al., 2009; Xie et al., 2009). Mu-delta density could also be affected in other pathological conditions enhancing delta receptor presence at the cell surface such as inflammatory pain conditions (Cahill et al., 2003) or voluntary alcohol consumption (van Rijn et al., 2012). In addition, expression of mu-α_2a_ heteromers in the nucleus of the solitary tract (NTS) is dynamically regulated and increased in hypertensive rats (Sun et al., 2015). In human peripheral blood mononuclear cells (PBMC), the mu agonist DAMGO induced CCR5 receptor synthesis through a TGFβ1 dependent mechanism (Happel et al., 2008), suggesting a role for mu-CCR5 heteromers in HIV1 entry in opiate abusers.

## Modulation of G protein signaling in native mu heteromers

**Figure 1:**
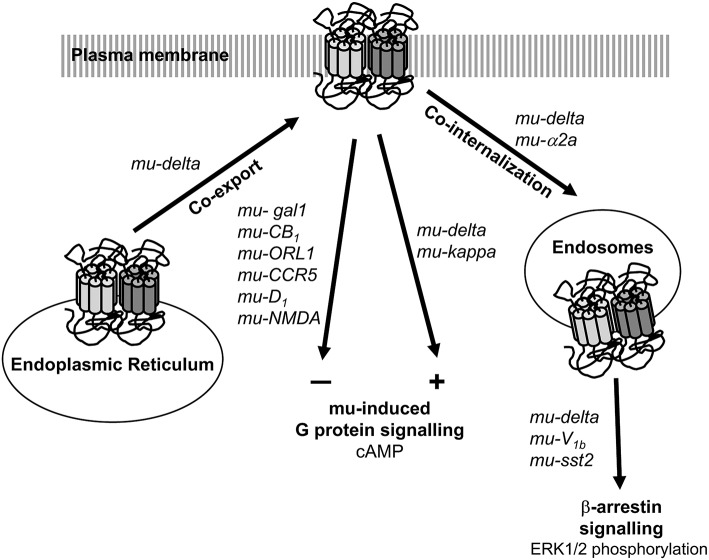
Impact of heteromerization on mu opioid receptor signaling and trafficking. Heteromerization can take place in the endoplasmic reticulum. Association with another opioid receptor positively modulates mu opioid receptor G protein-dependent signaling whereas association with a non-opioid receptor negatively regulates it. Heteromerization also favors the recruitment the β-arrrestin dependent pathway upon internalization in the endosomal compartments. Native receptor pairs for which information is available are indicated.

In SK-N-SH neuroblastoma cells co-expressing mu and delta receptors, occupancy of the binding site of one receptor by a non-signaling concentration of ligand increased binding and Gα signaling of the other receptor (Gomes et al., 2000, 2004, 2011). The nature of the first ligand did not seem important since agonist, antagonist or inverse agonist induced similar effects. Data therefore suggest that mu-delta heteromerization induces cross-allosteric modulation with a positive cooperativity promoted upon binding of the first ligand (Figure 1). Accordingly, co-application of the delta antagonist TIPPψ and mu agonist DAMGO or co-application of the mu antagonist CTAP and delta agonists deltorphin II or DPDPE increased hyperpolarization in a subset of neurons in the ventral tegmental area (VTA) (Margolis et al., 2017). Similarly, co-injection of subthreshold doses of the mu agonist DAMGO and the delta agonist deltorphin II in the rostral ventromedial medulla (RVM) of rats chronically treated with morphine increased γ aminobutyric acid (GABA)ergic inhibition through synergistic activation of the phospholipase A2 and cyclic adenosine monophosphate (cAMP)/protein kinase A (PKA) dependent pathways (Zhang and Pan, 2010). Moreover, mu-delta preferential coupling to the pertussis toxin insensitive Gαz subunit would not be desensitized by chronic morphine administration in the rat striatum and hippocampus (George et al., 2000; Kabli et al., 2014). Altogether, mu-delta positive crosstalk reinforces the inhibition of neuronal activity.

In contrast, heteromers formed with a non-opioid receptor appear to negatively modulate mu receptor G protein dependent signaling (Figure 1). In the VTA, co-activation of mu-Gal1 heteromers by galanin and endomorphin 1 decreased extracellular signal-regulated kinase ERK1/2, protein kinase B (AKT), and cyclic AMP response element binding protein (CREB) phosphorylation (Moreno et al., 2017). Accordingly, galanin could not prevent dopamine release promoted by local infusion of endomorphin 1 in the presence of an interfering peptide that disrupt mu-Gal1 physical interaction (Moreno et al., 2017). In addition, acute morphine administration enhanced ERK1/2 activation in the nucleus accumbens (Nacc) and amygdala of galanin knock-out mice compared to wild type mice (Hawes et al., 2008). These data suggest a negative crosstalk mediated by mu-Gal1 heteromers by which galanin dampens mu receptor signaling. In addition, the mu antagonist CTOP counteracted galanin induced ERK1/2, AKT and CREB phosphorylation indicative of a cross-antagonism on Gal1 receptor signaling (Moreno et al., 2017).

In BE(2)-C neuroblastoma cells co-expressing mu and ORL1 receptors, pretreatment with nociceptin decreased DAMGO potency and efficacy to inhibit adenylate cyclase (Mandyam et al., 2002). This effect was abolished in HEK293 cells co-transfected with receptor pairs unable to physically associate, which supports heteromer specificity (Wang et al., 2005). Additional examples of negative crosstalk on mu receptor signaling can be linked to heteromerization. Co-activation of mu and cannabinoid CB_1_ receptors by the mu agonist morphine and a non-signaling dose of the CB_1_ agonist WIN 55,212-2 decreased [^35^S]guanosine 5′-[γ-thio]triphosphate (GTPγS) binding and ERK1/2 signaling in SK-N-SH neuroblastoma cells (Rios et al., 2006) and Δ^9^-tetrahydrocannabinol (THC) allosterically decreased dihydromorphine binding at the mu receptor in rat striatal membranes (Vaysse et al., 1987). Similarly, co-activation of mu and adrenergic α_2a_ receptors decreased ERK1/2 phosphorylation in primary spinal cord neurons (Jordan et al., 2003). Also, the chemokine CCL5 induced phosphorylation of the mu receptor in human PBMC indicating cross-desensitization (Szabo et al., 2002). Finally, the dopamine D_1_ antagonist SCH233390 decreased G protein activation and ERK1/2 phosphorylation induced by the mu agonist DAMGO in mouse striatal membrane from wild type but not mice deficient for the D_1_ receptor (Tao et al., 2017). Activation of mu-NMDA heteromers by N-methyl-D-aspartate (NMDA) in the periaqueductal gray (PAG) also negatively regulated mu receptor activity by promoting PKA-dependent dissociation of the heteromer and subsequent mu receptor phosphorylation. This in turn promoted G protein uncoupling and receptor desensitization (Rodríguez-Muñoz et al., 2012).

Interestingly, constitutive activity has been reported for delta opioid (Costa and Herz, 1989), kappa opioid (Sirohi and Walker, 2015), ORL1 (Beedle et al., 2004), cannabinoid CB_1_ (Fioravanti et al., 2008), adrenergic α_2a_ (Pauwels et al., 2000), and mu opioid receptors (Wang et al., 2004). Since heteromers involving the mu receptor form in the ER in a ligand independent manner, receptor constitutive activity could represent an important determinant of the allosteric modulation and could contribute to the basal homeostasis of the cell in the absence of receptor stimulation. The release of endogenous peptides would however further modulate their functional impact because these receptors can still be activated by agonists (Canals and Milligan, 2008).

## Activation of β-arrestin signaling in native mu heteromers

In SK-N-SH neuroblastoma cells co-expressing mu and delta receptors, activation by the mu agonist DAMGO changed the spatio-temporal profile of ERK1/2 phosphorylation (Rozenfeld and Devi, 2007) (Figure 1). This was abolished in the presence of a β-arrestin 2 small interfering ribonucleic acid (siRNA) or in the presence of the delta selective antagonist TIPPψ (Rozenfeld and Devi, 2007) suggesting that activation of heteromers involving the mu receptor can promote β-arrestin dependent signaling. Mice deficient for β-arrestin 2 developed less tolerance to morphine (Bohn et al., 2002), data thus suggest that the recruitment of the β-arrestin pathway by mu heteromers contributes to morphine tolerance. This hypothesis is also supported by the observation that tolerance to morphine develops more slowly in mice deficient for the vasopressin V_1B_ receptor or in the presence of a V_1B_ selective antagonist (Koshimizu et al., 2018). In the mouse RVM, vasopressin V_1B_ receptors constitutively associate with β-arrestin 2 through a leucine rich motif present in the V_1B_ C-terminus (Koshimizu et al., 2018). This suggests that physical association with the V_1B_ receptor facilitates the recruitment of the β-arrestin pathway by the mu receptor, and contributes to the development of morphine tolerance. Accordingly, removal by genome editing with the clustered regularly interspaced short palindromic repeats-CRISPR associated protein 9 (CRISPR-Cas9) system of the leucine rich motif responsible for the receptor V_1B_-β-arrestin interaction increased morphine-induced analgesia and reduced adenylate cyclase supersensitization and morphine-induced tolerance and physical dependence (Koshimizu et al., 2018).

Of note, co-activation of mu and somatostatin sst2 receptors by dermorphin and L-054,264 in pancreatic cancer lines similarly altered the spatio-temporal profile of ERK1/2 phosphorylation, potentiating the epithelial to mesenchymal transition (Jorand et al., 2016).

## Receptor co-internalization in native mu heteromers

Co-internalization of endogenous heteromers is less documented largely due to the lack of appropriate tools (Figure 1). Receptor internalization contributes to desensitize G protein dependent signaling and favors β-arrestin dependent signaling (Calebiro et al., 2010). Accordingly, co-internalization of mu and adrenergic α_2a_ receptors was dependent on β-arrestin 2 recruitment and mitogen-activated protein kinase (MAPK) p38 activation in the mouse DRGs (Tan et al., 2009).

Mu-delta co-internalization was observed following activation by the mu-delta biased agonist CYM51010 in primary hippocampal neurons from fluorescent double knock-in mice (Derouiche et al., 2018) but could not be detected in the spinal cord following SNC80 application (Wang et al., 2018). Since SNC80 promoted mu-delta co-internalization in co-transfected HEK293 cells (He et al., 2011), this observation highlights the influence of the cellular environment.

## Mu heteromers modulate nociception, morphine analgesia and tolerance

Several observations support the implication of mu heteromers in the control of the nociceptive threshold. The lower response to inflammatory or chemical stimuli in CCR5 knock-out mice or upon injection of a CCR5 antagonist indicate that mu-CCR5 heteromers contribute to dampen the basal nociceptive threshold by exerting a negative crosstalk on mu receptor signaling (Lee et al., 2013). Mapping mu and delta receptors in the central and peripheral nervous systems using double fluorescent knock-in mice revealed mu-delta co-expression in discrete neuronal populations located in networks involved in the perception and processing of nociceptive stimuli (Erbs et al., 2015). Accordingly, disrupting mu-delta physical interaction with an interfering peptide in naïve mice increased morphine-induced thermal analgesia (He et al., 2011). In rats chronically treated with morphine or with persistent inflammatory pain, co-administration of low doses of mu and delta agonists in the RVM enhanced mechanical and thermal analgesia (Sykes et al., 2007; Zhang and Pan, 2010). Since delta receptor expression is increased in both pathological conditions (Cahill et al., 2003; Gendron et al., 2015; Ong et al., 2015; Erbs et al., 2016), this synergistic effect can be explained by the positive crosstalk at mu-delta heteromer elicited by receptor co-activation (Gomes et al., 2000, 2004, 2011). Similarly, co-activation by morphine and the subsequently released dynorphin 1–17 acted synergistically at mu-kappa heteromers to increase spinal morphine analgesia (Chakrabarti et al., 2010). However, this effect is sex-dependent and more pronounced in proestrous female mice where mu-kappa heteromers are most abundant (Chakrabarti et al., 2010; Liu N. J. et al., 2011).

Activation of mu-delta heteromers by the mu agonist DAMGO (Rozenfeld and Devi, 2007) or co-activation of mu-V_1b_ heteromers by vasopressin and morphine (Koshimizu et al., 2018) increased β-arrestin 2 recruitment and signaling. Importantly, this pathway participates to the development of morphine tolerance (Bohn et al., 2000), which suggests a contribution from mu heteromers. Accordingly, disruption of the physical contact between the mu and delta opioid receptors (Xie et al., 2009; He et al., 2011) or between the mu and vasopressin V_1b_ receptors (Koshimizu et al., 2018) decreased morphine tolerance. In addition, activation of mu-NMDA heteromers in the PAG reduces morphine efficacy through a dual mechanism. Indeed, stimulation by NMDA decreases the analgesic effect of morphine by exerting a negative crosstalk on mu signaling whereas morphine binding to the mu receptor potentiates the NMDA-Ca^2+^/calmodulin-dependent protein kinase (CAMKII) pathway and contributes to morphine tolerance (Rodríguez-Muñoz et al., 2012).

Other roles for mu heteromers include morphine-induced itch generated by cross-activation of the GRPR signaling in MORD1-GRPR heteromers (Liu X. Y. et al., 2011). Moreover, mu-α_2a_, mu-CB_1_, or mu-ORL1 heteromers very likely represent additional key players since all four receptors modulate nociception but, to date, a direct link to heteromerization with the mu receptor is still lacking.

## Mu heteromers modulate reward processing and addiction to opioid drugs

Modulation of mu receptor signaling by heteromer formation in the mesocorticolimbic pathway is bound to have a profound impact on the rewarding properties of opioid drugs and natural stimuli. Accordingly, galanin-dependent dampening of opiate reinforcing and rewarding properties was abolished upon disruption of mu-Gal1 heteromers in the VTA (Moreno et al., 2017) or in galanin knock-out mice (Hawes et al., 2008). Also, chronic morphine treatment increased mu-delta heteromer expression in several brain regions including the VTA and Nacc (Gupta et al., 2010). Therefore, the positive cross talk at mu-delta heteromers observed in a subset of VTA neurons could contribute to increased dopamine release in the Nacc and opiate reinforcing properties (Margolis et al., 2017).

Also, systemic injection of the endocannabinoid 2-arachidonoyl (2-AG) hydrolysis inhibitor JZL184 increased the concentration of the endogenous ligand and enhanced social play behavior in adolescent rodents (Manduca et al., 2016). This effect was blocked by infusing the mu antagonist CTAP in the Nacc and was absent in mu receptor knock-out mice (Manduca et al., 2016). Reciprocally, systemic injection of the mu agonist morphine increased social play and was abolished by the CB_1_ antagonist SR1417-16 or in CB_1_ receptor knock-out mice (Manduca et al., 2016). This bidirectional cross-antagonism suggests that mu-CB_1_ heteromers in the Nacc modulate the strong rewarding value of social play.

Mu receptors are also involved in other aspects of opiate addiction such as locomotor sensitization and could achieve their modulatory control through heteromerization with dopamine D_1_ receptors. Indeed, opiate hyperlocomotion and locomotor sensitization were abolished in dopamine D_1_ receptor in knock-out mice or following local injection of the D_1_ antagonist SCH23390 in the Nacc (Tao et al., 2017).

## Mu heteromers modulate anxiety and depression

Pharmacological and knock-out based studies linked an anxiogenic and depressant phenotype to mu receptor activation and, on the opposite, associated an anxiolytic and antidepressant phenotype with delta receptor activation (Lutz et al., 2014).

Systemic administration or local micro-infusion in the Nacc of the delta agonist UFP512 promoted anxiolytic- and anti-depressant-like activity (Vergura et al., 2008; Kabli et al., 2013). These effects were abolished by pretreatment with the mu antagonist CTOP or the delta antagonist naltrindole or following disruption of mu-delta physical contact in the Nacc (Kabli et al., 2013). These data therefore suggest that accumbal mu-delta heteromers participate to the modulation of anxio-depressive states.

## Mu heteromers modulate metabolic disorders

Mu receptors are known to control autonomous functions. Higher levels of mu-α_2a_ heteromers in the NTS were correlated with increased blood pressure in hypertensive rats (Sun et al., 2015). In normotensive rats, mu-α_2a_ heteromerization induced by the mu agonist DAMGO was paralleled by increased blood pressure. Treatment with the mu antagonist CTAP antagonized DAMGO changes in normotensive rats and reduced mu-α_2a_ heteromerization and blood pressure in hypertensive rats (Sun et al., 2015). Thus, activation of the mu receptor by endogenous opioid peptides dampens the activity of the α_2a_ adrenergic receptors thereby potentiating hypertension.

Interactions between mu and somatostatin receptors have been postulated to influence tumor cell growth (Hatzoglou et al., 2005). Recently, mu-sst2 heteromers were identified in pancreatic cancer lines and in tissue from patients with pancreatic ductal adenocarcinoma. Co-activation of the receptors initiated the epithelial to mesenchymal transition, which is associated with increased metastatic potential (Jorand et al., 2016).

## Mu heteromers as a novel therapeutic target

The bivalent ligand MDAN-21 composed of the mu agonist oxymorphone and the delta antagonist naltrindole tethered by a 21 amino acid long linker was developed to selectively target mu-delta heteromers (Daniels et al., 2005). The length of the linker was designed to enable simultaneous binding of the two ligand moieties to the orthosteric binding pockets of two GPCRs in physical contact. MDAN-21 induced analgesia with low tolerance, low physical dependence and no reinforcing properties (Daniels et al., 2005; Lenard et al., 2007; Aceto et al., 2012) providing a proof of concept that selective targeting of mu-delta heteromers may represent a valid therapeutic strategy, in particular for patients on opiate maintenance treatment.

More recently, the bivalent ligand MCC22 linking the mu agonist oxymorphone to the CCR5 antagonist TAK220 has been proposed to inhibit inflammatory and neuropathic pain by targeting mu-CCR5 heteromers (Akgün et al., 2015). This is in line with the enhanced nociception observed in CCR5 receptor knock-out mice or in the presence of a CCR5 antagonist (Lee et al., 2013).

A major limitation to the therapeutic use of bivalent ligands is their poor capacity to cross the blood brain barrier (Le Naour et al., 2013; Jörg et al., 2015). Therefore, monovalent bifunctional ligands that would selectively target mu heteromers have been developed (Schiller, 2010; Günther et al., 2018). Eluxadoline is a mixed mu agonist delta antagonist recently been approved by the FDA for the treatment of the irritable bowel syndrome (FDA application N°206940). Arguments in favor of binding to mu-delta heteromers include lower efficacy in mice deficient for the delta receptor and reduced signaling in the presence of mu-delta selective antibodies (Fujita et al., 2014b). Eluxadoline thus represents the first drug on the market designed to target heteromers. In preclinical models, other ligands further support preferential activation of mu heteromers as a valuable therapeutic approach. The mu-delta biased agonist CYM51010 induced potent thermal analgesia comparable to morphine but less tolerance and physical dependence (Gomes et al., 2013) and the mu-kappa agonist NNTA produced strong analgesia devoid of tolerance, physical dependence, or reinforcing properties upon intrathecal injection in mice (Yekkirala et al., 2011).

## Conclusion

Our current appreciation of the role of mu heteromer is still in its infancy and their contribution to mu receptor-dependent behavior likely underestimated. So far, physical proximity has only been validated for a limited number of receptor pairs *in vivo* and their functional interactions addressed in a handful of tissue or brain areas. Moreover, heteromer expression is dynamically regulated depending on physiopathological conditions. No doubt that both novel functions and receptor pairs will be uncovered in the future, which further emphasizes their potential as innovative therapeutic targets.

## Author contributions

All authors listed have made a substantial, direct and intellectual contribution to the work, and approved it for publication.

### Conflict of interest statement

The authors declare that the research was conducted in the absence of any commercial or financial relationships that could be construed as a potential conflict of interest.
